# Massive Expansion of Ubiquitination-Related Gene Families within the *Chlamydiae*

**DOI:** 10.1093/molbev/msu227

**Published:** 2014-07-28

**Authors:** Daryl Domman, Astrid Collingro, Ilias Lagkouvardos, Lena Gehre, Thomas Weinmaier, Thomas Rattei, Agathe Subtil, Matthias Horn

**Affiliations:** ^1^Department of Microbiology and Ecosystem Science, University of Vienna, Vienna, Austria; ^2^Unité de Biologie des Interactions Cellulaires, Institut Pasteur, Paris, France

**Keywords:** gene families, birth and death model, intracellular bacteria, effector proteins, F-box

## Abstract

Gene loss, gain, and transfer play an important role in shaping the genomes of all organisms; however, the interplay of these processes in isolated populations, such as in obligate intracellular bacteria, is less understood. Despite a general trend towards genome reduction in these microbes, our phylogenomic analysis of the phylum *Chlamydiae* revealed that within the family Parachlamydiaceae, gene family expansions have had pronounced effects on gene content. We discovered that the largest gene families within the phylum are the result of rapid gene birth-and-death evolution. These large gene families are comprised of members harboring eukaryotic-like ubiquitination-related domains, such as F-box and BTB-box domains, marking the largest reservoir of these proteins found among bacteria. A heterologous type III secretion system assay suggests that these proteins function as effectors manipulating the host cell. The large disparity in copy number of members in these families between closely related organisms suggests that nonadaptive processes might contribute to the evolution of these gene families. Gene birth-and-death evolution in concert with genomic drift might represent a previously undescribed mechanism by which isolated bacterial populations diversify.

## Introduction

The genomes of organisms reveal complex histories of gene transfer, loss, gain, and rearrangement. The extent that these processes play in shaping gene families of both prokaryotes and eukaryotes are markedly different. Gene gain within eukaryotes is largely driven by intragenomic duplication events (Lynch and Conery 2000; Koonin et al. 2002; Yang et al. 2003; Kaessmann 2010), and although duplication certainly shapes bacterial genomes, most gains are the result of horizontal gene transfer (HGT) events (Ochman et al. 2000; Lerat et al. 2005; Treangen and Rocha 2011). Estimates of the contribution gene duplication processes play across domains of life vary from 65% to 30% in the genomes of *Arabidopsis* and *Escherichia coli,* respectively (Zhang 2003)*.* Although genetic innovation typically arises through gene acquisition from foreign sources, gene duplication events are increasingly being recognized as an important driver of bacterial genome evolution (Goldman et al. 2006; McLeod et al. 2006; Cho et al. 2007).

Comparisons of closely related organisms have revealed a highly dynamic landscape of gene families, in which the copy number between species can vary substantially (Pushker et al. 2004; Lerat et al. 2005). Given this background, an intriguing evolutionary backdrop to study gene family evolution is within obligate, intracellular bacteria. In these populations, the fixation of mutations is strongly affected by genetic drift, with a propensity in these genomes for deletion (Kuo and Ochman 2009a), and thus gene family expansions within these genomes are generally rare (Hooper and Berg 2003; Gevers et al. 2004). Insightful analysis on gene family evolution is best approached when comparing multiple genomes from closely related species, facilitating identification of paralogs (homologous genes resulting from duplication), orthologs (homologous genes resulting from speciation), or xenologs (homologous genes derived from HGT). In this regard, the phylum *Chlamydiae* offers an ensemble of fully sequenced genomes across multiple families.

All members of the phylum *Chlamydiae* are obligate, intracellular bacteria and represent one of the most ancient and successful lineages associated with eukaryotes (Horn 2008; Subtil et al. 2014). These organisms all share a characteristic biphasic developmental cycle consisting of an infectious, extracellular state and an intracellular replicative state. The phylum can be divided into two major phylogenetic groupings: The family Chlamydiaceae, which encompass well known animal and human pathogens such as *Chlamydia trachomatis* and *C. pneumoniae,* and a group of families comprising the environmentally distributed chlamydiae such as Simkaniaceae*,* Waddliaceae*,* and Parachlamydiaceae collectively referred to as environmental chlamydiae. Recently, it was shown that the diversity of the phylum is tremendously greater with perhaps over 200 families spanning nearly every environment (Lagkouvardos et al. 2013). All members of the *Chlamydiae* show notable genomic reductions and truncated metabolic pathways including the inability to synthesize many amino acids and nucleotides (Stephens et al. 1998; Kalman et al. 1999; Horn et al. 2004; Bertelli et al. 2010; Collingro et al. 2011; Myers et al. 2012).

In this study, we set out to determine how gene families have evolved in members of the phylum *Chlamydiae*. We present four new genome sequences for members of the family Parachlamydiaceae, which include two genome sequences for the genus *Neochlamydia*. We show that organisms within the Parachlamydiaceae have unprecedented numbers of proteins harboring domains typically found in eukaryotes, the majority of which are related to eukaryotic ubiquitination pathways. We show that these genes have undergone rapid expansions and form the largest gene families within the phylum. We demonstrate that many of these large gene families are evolving under a gene-birth-death model (Nei and Rooney 2005) and that differences between closely related organisms may be explained by genomic drift.

## Results

### Genome Sequencing of Novel Members of the Chlamydiae

**Fig. 1.**
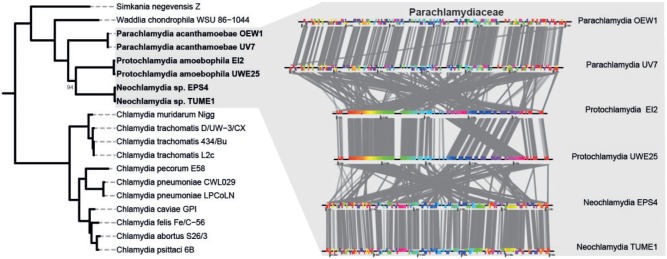
Phylogeny of the *Chlamydiae* and rearrangement history of genomes within the Parachlamydiaceae. Phylogeny of the *Chlamydiae* based on 32 phylogenetic marker proteins. A Bayesian analysis using MrBayes (Ronquist and Huelsenbeck 2003) was performed on a set of 24 ribosomal proteins in addition to GyrB, RecA, RpoB, RpoC, and EF-Tu from 19 sequenced members of the phylum (supplementary table S2, Supplementary Material online). Members of the Planctomycetes (*Blastopirellula marina* DSM 3645, *Candidatus* Kuenenia stuttgartiensis, and *Gemmata obscuriglobus* UQM 2246) and the Verrucomicrobia (*Akkermansia muciniphila* MucT, *Lentisphaera araneosa* HTCC2155, *Opitutus terrae* PB90-1, and *Verrucomicrobium spinosum* DSM 4136) were used as outgroups (not shown). Colors denote family level classification. Posterior probability scores are indicated only if below 100%. To the right, conserved synteny and rearrangement history of genomes within the Parachlamydiaceae are shown. The genomes of six members of the family were aligned using MAUVE to elucidate synteny between genomes and visualized using genoPlotR. Extensive rearrangements are apparent between members of different genera, whereas within genus, comparisons show little rearrangements, with a notable exception in the *Protochlamydia* where a large block has been rearranged.

Currently, there are nine described families within the *Chlamydiae*; however, the majority of available genome sequences come from a single family, the pathogenic Chlamydiaceae. To deepen our insights into a family outside of the Chlamydiaceae, we sequenced the genomes of four members of the family Parachlamydiaceae, which include two members of the genus *Neochlamydia*, and two additional genomes of *Protochlamydia* and *Parachlamydia*. All of the newly sequenced Parachlamydiaceae members were isolated from free-living amoeba. With the exception of *Neochlamydia* sp. EPS4, the isolates have been described previously (Fritsche et al. 2000; Heinz et al. 2007; Schmitz-Esser et al. 2008). The draft genomes represent nearly complete genome sequences based on paired end read data (90–96%) and the presence of conserved single-copy marker genes (98–100%; supplementary table S1, Supplementary Material online). Using these additional genome sequences, we first aimed to construct a phylogenetic framework of the phylum *Chlamydiae* using concatenated alignments of 32 marker proteins (supplementary table S2, Supplementary Material online). Phylogenetic trees obtained with different methods confirmed the monophyly of the Chlamydiaceae and the Parachlamydiaceae with strong support (fig. 1). The Chlamydiaceae can be subdivided in two previously recognized groups, and within the Parachlamydiaceae, the genera *Protochlamydia*, *Neochlamydia*, and *Parachlamydia* were recovered with high confidence.

All members of the Chlamydiaceae show highly similar genomes in terms of gene content and synteny (Myers et al. 2012); however, between chlamydial families, rearrangements have played a major role in genome evolution (Collingro et al. 2011). Whole-genome alignments of members of the Parachlamydiaceae clearly illustrate that within the genera *Protochlamydia*, *Neochlamydia*, and *Parachlamydia,* there are few rearrangements, and the genomes are highly syntenic (fig. 1). Between these genera, however, there have been extensive genome rearrangements demonstrating the surprising dynamic nature of these reduced genomes.

### The Gene Family Landscape of the Chlamydiae

**Fig. 2.**
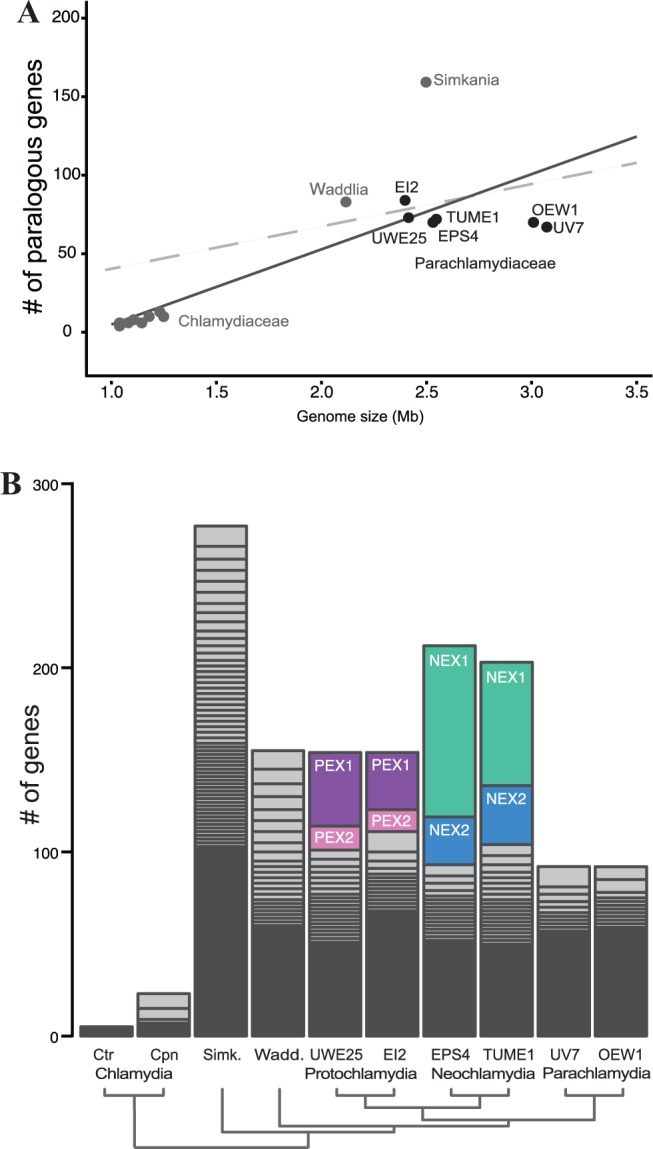
The paralogous gene landscape of the *Chlamydiae.* (*A*) Number of paralogous genes within the *Chlamydiae.* The number of paralogous genes, not including multiple copies, is plotted against genome size along with a linear regression line (*y* = 47.87*x* – 42.9; *R*^2 ^= 0.70; black line). The dashed gray line is plotted as a reference from 200 prokaryotic genomes (Bratlie et al. 2010). (*B*) Distribution of chlamydial gene families per genome with two or more members. The number of genes within each family is plotted for representative genomes. The genomes of the Chlamydiaceae have relatively small gene family sizes. The polymorphic outer membrane proteins comprise the largest gene families in the Chlamydiaceae and can be seen as the two largest blocks in the *Chlamydia pneumoniae* LPCoLN (Cpn) bar. The size distribution of gene families is ordered from smallest to greatest, and the appearance of a solid “black box” at the base is merely an effect of the spacing of many small gene families. There are several extensive gene families (labeled) within members of *Neochlamydia* (NEX1, NEX2) and *Protochlamydia* (PEX1, PEX2).

To explore gene family evolution among members of the *Chlamydiae*, we first identified gene families using clusters of orthologous groups of proteins within the predicted proteomes from 19 chlamydial genomes (supplementary table S2, Supplementary Material online). We then searched for gene families that contain expansion events, that is, those having multiple members from one organism. Previous work has demonstrated that genome size correlates with the number of paralogs, with larger genomes containing more paralogs than smaller ones (Bratlie et al. 2010). Taking into account all gene family members, regardless of whether they originate from duplication processes or transfer events, this trend is generally observed within chlamydial genomes (fig. 2*a*).

As described previously, gene family expansions are sparse within the genomes of the Chlamydiaceae (Kalman et al. 1999; Kamneva et al. 2012), with *C. pneumoniae* CWL029 harboring the largest number (*n* = 24). In line with previous observations, the largest gene families identified in our study encode the polymorphic membrane proteins (PMPs) (Grimwood and Stephens 1999; Gomes et al. 2006) including nine members from *C. pneumoniae* LPCoLN and two from the *C. trachomatis* serovars. The observed split of PMPs among several smaller gene families in our analysis is an indication that our approach is rather conservative in assigning a protein to a gene family.

The total number of expansion events (*n* = 277) detected in the genome of *Simkania negevensis* represents a 10-fold increase when compared with the Chlamydiaceae. As these group into many small gene families, the extended number of gene copies in *S. negevensis* is the result of many small-scale duplication or transfer events (fig. 2*b*). This situation is similar in *Waddlia chondrophila*. In stark contrast, roughly half of the total of genes resulting from expansion events in *Neochlamydia* and *Protochlamydia* are the contribution of only few gene families.

### Large Gene Family Expansions in the Parachlamydiaceae

The detection of large gene families in *Neochlamydia* and *Protochlamydia* indicates that there have been several large-scale expansion events within the Parachlamydiaceae. Notably, different gene families are expanded in *Neochlamydia* and *Protochlamydia* (fig. 2*b*). These represent the four largest gene families (containing between 27 and 138 members) found within the phylum and include two gene families specific to *Neochlamydia* and two restricted to *Protochlamydia*.

Intrigued by these four large-scale lineage-specific expansion events between the species pairs of *Protochlamydia* and *Neochlamydia*, we sought to better characterize these gene families, as most of their members are yet unknown with respect to their functional role (i.e., they are classified as hypothetical proteins). Remarkably, despite being in different gene families from different organisms, there are several similarities between these proteins. Firstly, they all encompass protein–protein interaction domains such as leucine-rich repeats (LRRs) or tetratricopeptide repeats (TPRs). Secondly, many of them contain additional domains typically found in eukaryotes, such as F-boxes, BTB-boxes, and RING/U-boxes, which are associated with eukaryotic ubiquitination pathways (Angot et al. 2007).

### Eukaryotic Ubiquitination-Associated Domains Predominate Large Gene Families

**Fig. 3.**
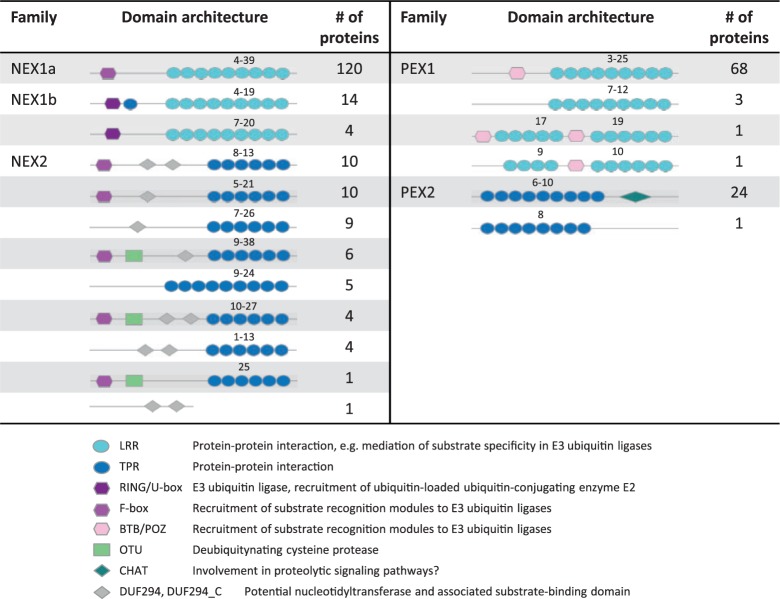
Protein domain architecture of largest gene families. The domain architecture of *Neochlamydia* (NEX1, NEX2) and *Protochlamydia* (PEX1, PEX2) gene families are shown. The range of the number of domain repeats and functional assignments of the detected domains are indicated. NEX1 can be divided into two subfamilies based on phylogeny and domain presence/absence. A role of these proteins in the context of eukaryotic cells can be postulated based on the presence of domains otherwise found in eukaryotes.

The largest gene family, termed *Neochlamydia expansion* 1 (NEX1), in the phylum comprised a total of 138 members, which are contributed by the two *Neochlamydia* genomes*.* The domain architecture within this large gene family is heterogeneous; however, all proteins contain various C-terminal repetitions of LRR domains (fig. 3). We have identified two subfamilies that we delineate NEX1a and NEX1b within the NEX1 family. The majority of members fall into the NEX1a subfamily, in which they have a highly conserved N-terminal F-box or F-box-like domain. The members have an average of 77% sequence similarity among each other, and the F-box-like domain is 57% and 45% similar to *Acanthamoeba castellanii* and human F-box-like domains, respectively. The smaller NEX1b family, in contrast, has a conserved RING/U-box at the N-terminus. Both the F-box and RING/U-box domains are associated with eukaryotic E3 ubiquitin ligase complexes (Willems et al. 2004). Between all members in the NEX1 family, there is a region of roughly 50 amino acids between the predicted N-terminal domains and the LRRs that is highly conserved. However, we failed to detect any known domains in this region nor was there any homology to known proteins.

The second large gene family within the *Neochlamydia* represents the third largest family of the phylum. This large gene family, termed NEX2, comprised 50 proteins (fig. 3). Similar to NEX1, the prevailing domain architecture is that of eukaryotic-like E3 ubiquitin ligase-associated domains paired with repeat domains. This family is defined by the presence of multiple TPR domains at the C-terminus, and a general conservation of an F-box domain at the N-terminus in the majority of members. In most members, there is also a conserved DUF294 domain located mid protein, which is a putative nucleotidyltransferase. In ten members, there is an ovarian tumor (OTU) (Balakirev et al. 2003) domain directly following the F-box domain followed by the DUF294 domain.

The other large gene families occur primarily in the members of the *Protochlamydia* and represent the second and fourth largest gene families of the phylum. The largest gene family in the *Protochlamydia* (PEX1) comprised 73 members in total (fig. 3). Intriguingly, another E3 ubiquitin ligase-related domain, the BTB domain, is present in all but three members, at the N-terminus. The BTB domain is then coupled to C-terminal LRR domains in all members. The PEX2 family comprised a total of 27 proteins that, despite no detectable domain at the N-terminus, share multiple TPR domains in the middle of the protein followed by a CHAT domain (Sakakibara and Hattori 2000) at the C-terminus.

In summary, the four largest gene families represent a surprisingly diverse armada of proteins, which most likely function within eukaryotic host cells where they potentially interfere with the ubiquitination pathway. The heterogeneity in domain architecture among these proteins interestingly mirrors that of their eukaryotic counterparts (Perez-Torrado et al. 2006; Xu 2006).

### A Pool of Putative Effector Proteins

If the members of the largest gene families in the phylum *Chlamydiae* serve as effector proteins for host manipulation, they would need to be secreted and transported to the host cell cytosol. This is typically achieved through a type III secretion system, a well-conserved virulence mechanism among the *Chlamydiae*, which has been shown to translocate several characterized effectors (Peters et al. 2007; Betts et al. 2009). Indeed, many of the proteins found within the expanded *Neochlamydia* and *Protochlamydia* gene families are predicted by computational analysis to be secreted by the type III secretion system and to be extracellular, host associated. Within the NEX1 family, 66 of 138 (49%) members are predicted to be secreted. A total of 37 (51%) and 10 (37%) were predicted to be secreted from within the PEX1 and PEX2 gene families, respectively. The NEX2 gene family had the fewest predicted with only two members.

As the identification of the signal for secretion via the type III secretion system is inherently difficult (Arnold et al. 2009), we tested representatives of each of the four largest gene families in vitro using a heterologous type III secretion substrate assay with *Shigella flexneri* as a host for protein expression. This assay has been used to successfully characterize type III secretion effector proteins from the chlamydiae before (Subtil et al. 2001), and because of the lack of routine genetic tools, the *Sh. flexneri* system is an attractive surrogate method for analyzing type III secretion in chlamydiae in vivo. This experiment demonstrated that the tested members of NEX1, NEX2, PEX1, and PEX2 contain a functional type III secretion recognition signal (supplementary fig. S1, Supplementary Material online). Together with the presence of eukaryotic-like domains and computational predictions, this strongly indicates that these gene families are large pools of effector proteins.

### Molecular Evolution of Large Gene Families

To better understand how these large gene families may have evolved, we reconstructed their phylogenetic relationships. Gene family trees were calculated using conserved sites among the protein alignment (supplementary figs. S2–S6, Supplementary Material online). The average amino acid identity between members ranges from 45% to 64%, with the most closely related sequences belonging to PEX2. Tree topologies suggest that the members of all four gene families have rapidly diverged as indicated by their long branch lengths. Although the number of LRR and TPR domains varies dramatically between 1 and 39, this had no apparent effect on the phylogenetic placement.

We find clear cases in which the orthologs of two species group together, indicating expansions have occurred before speciation (supplementary figs. S2–S6, Supplementary Material online). Alternatively, expansions post speciation is apparent in all gene families. Reconciliation of gene family trees with the species tree indicates that, in addition to expansions, many gene losses have occurred for each gene family (supplementary table S3, Supplementary Material online). For instance, for the NEX1 family, there have been 78 expansion events, whereas 33 losses have occurred, attributed to 13 and 20 losses in *Neochlamydia* spp. EPS4 and TUME1, respectively. There were nearly equal losses between EPS4 and TUME1 (13 and 10) in NEX2 and a total of 46 expansions. Similarly, PEX1 consists of 52 expansions, and 15 and 9 losses in *Protochlamydia amoebophila* EI2 and UWE25, respectively.

### A Birth-and-Death Model of Evolution

A pattern of differential gain, loss, and maintenance of gene family members is strongly indicative of these gene families evolving according to a birth-and-death model (Nei and Rooney 2005). Because of this differential maintenance of gene family members, the hallmarks of the birth-and-death model are interspecies clustering of members in the phylogenetic trees and the presence of pseudogenes from degraded members (Nei 2007). As we observe interspecies clustering for the PEX and NEX gene families (supplementary figs. S2–S6, Supplementary Material online), we also tested for pseudogenization events in the intergenic regions of the *P. amoebophila* UWE25 genome (the draft *Neochlamydia* genomes are less suitable for this analysis). By utilizing BLAST, we searched for matches to the predicted proteome using all intergenic regions as a query. We then mapped the best BLAST hits, representing 116 pseudogenes, to their respective gene families to get a picture of a given families’ representation in the intergenic regions. The most represented gene family in the intergenic regions, surprisingly, was PEX1 (16 pseudogenes). For PEX2, one pseudogenized fragment was detected. The observed presence of interspecies clustering and pseudogenes, and the dynamic history of gains and losses within the gene families are indicative of a birth-and-death model of evolution.

In contrast, if these families were evolving via concerted evolution, the phylogenetic trees would depict intraspecies clustering, that is, that members of a gene family will be more homologous to the other members from the same organism than to that of other species. Intraspecies clustering occurs due to repeated recombination among gene family members within a genome, leading to an overall high sequence similarity of all members, a process known as gene conversion (Santoyo and Romero 2005). We thus tested for the possibility of recombination within the gene families using the methods implemented in the RDP4 software suite (Martin et al. 2010). Care must be taken, however, when assessing the impact of recombination among divergent proteins, as the recombination signal is quite error prone when proteins share less than 70% similarity, and these methods are heavily dependent on the alignment (Martin et al. 2010). We detect some recombination events between members within the PEX and NEX gene families; however, the majority of the predictions are only marginally significant (the consensus scores are below the confidence threshold of 0.6). In the NEX1a family, the portion of the sequences most identified as recombinant is the F-box domain (supplementary fig. S8, Supplementary Material online). This should be judiciously interpreted, as this might represent sequence similarity due to purifying selection operating on this domain. Overall, we did not find convincing evidence that recombination has played a major role in shaping the evolution of the PEX and NEX gene families.

### Gene Duplications and Purifying Selection

**Fig. 4.**
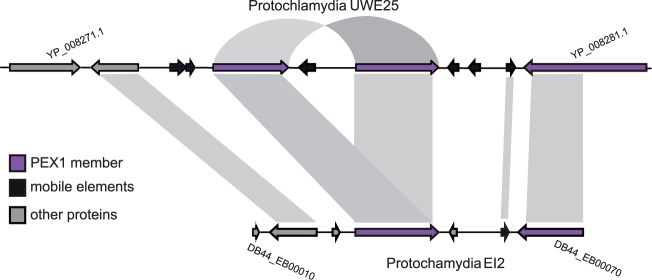
Example of duplication of BTB-box proteins in the *Protochlamydia*. A new duplicate has arisen in *Protochlamydia amoebophila* UWE25 (shown via the arrow) after the split from *P. amoebophila* EI2. BTB-box proteins are indicated in purple. The phylogenetic placement of these proteins (supplementary fig. S5, Supplementary Material online) supports this scenario. Orthologous proteins between the *Protochlamydia* are indicated by connecting blocks.

Gene family expansions can be the result of either gene duplication or HGT. The disentangling of these events is not trivial and, in fact, may be impossible in the case of the gene families investigated here due to lineage-specific evolution and the absence of clear homologs in other bacterial taxa (Kuo and Ochman 2009b). However, several lines of evidence suggest that, regardless of the initial origin of these genes, gene duplication processes have played a clear role in the evolution the large Parachlamydiaceae gene families. First, a hallmark of gene duplication is the presence of tandem arrays of gene copies. In this regard, we find several large tandem arrays with members of the large gene families in *Protochlamydia* and *Neochlamydia*, including examples for recent duplications (fig. 4, supplementary fig. S7, Supplementary Material online). In the *P. amoebophila* UWE25 genome, we detected 47 tandem duplication events (distance within 10 genes) represented by only 13 gene families. Nearly half of these are the contribution of PEX1 (*n* = 20); however, they appear in several clusters spread throughout the genome. PEX2 demonstrates the most dramatic case of a tandem array consisting of 13 members. After the PEX gene families, the third largest tandem array only comprised three members, intriguingly also F-box domain containing proteins. NEX1 has 50 instances (36%) where members are found within five genes of each other. Second, the majority of the large gene family members meet the generally used criterion for identification of paralogs, that is, they show at least 30% amino acid sequence identity over at least 60% of the protein length. Thirdly, phylogenetic trees show several species-specific expansion events (including those genes still arranged in tandem arrays), which are best explained by gene duplication.

Effector proteins have been shown to be among the fastest evolving proteins in a number of pathogen genomes (Nogueira et al. 2012) and are often shown to be under positive selection. To assess the mode of selection acting on members of the expanded gene families, we calculated the ratio of nonsynonymous (d*N*) versus synonymous (d*S*) substitution rates for each of the four gene families. A d*N*/d*S* ratio equal to 1 indicates a neutral state of selection, whereas values higher or lower than 1 indicate positive or purifying selection, respectively. Global estimates of d*N*/d*S* under the Model M0 from CodeML (Yang 2007) ranged from 0.29 (NEX2) to 0.63 (NEX1a). Additionally, using pairwise sequence comparisons for d*N*/d*S* calculation, we did not find support that these gene families are currently evolving under positive selection. However, the probability of these gene families evolving under purifying selection was highly significant (*P* < 0.01). Therefore, purifying selection is currently the dominant force driving the evolution of these gene families and thus facilitates their maintenance.

### Massive Expansion of Ubiquitination-Associated Proteins

**Fig. 5.**
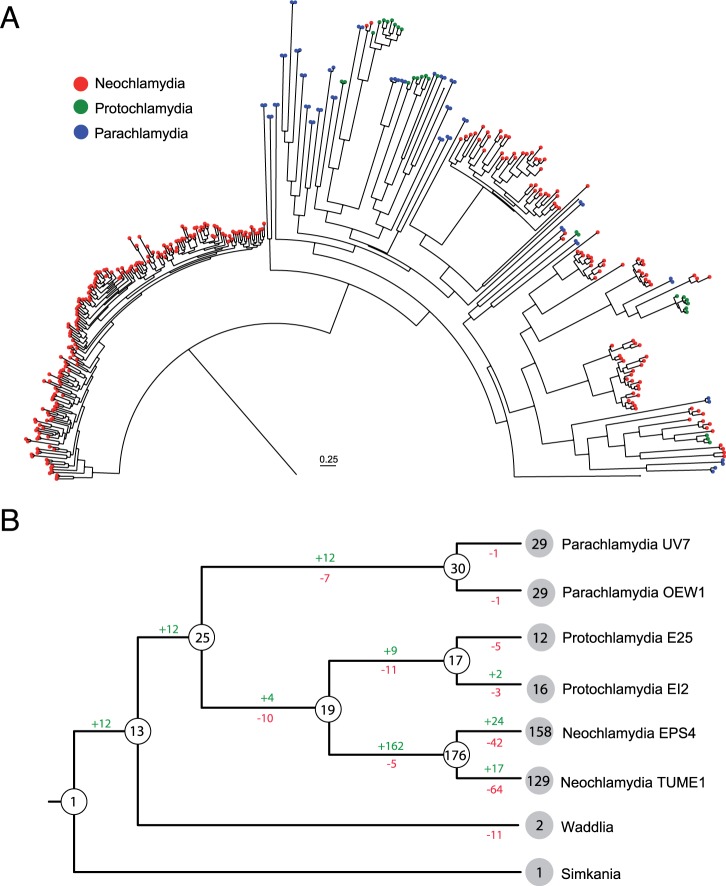
The phylogeny and evolutionary history of the F-box superfamily within the *Chlamydiae*. (*A*) The phylogeny of 376 proteins within the *Chlamydiae* that harbor an F-box/F-box-like domain. This domain was extracted from each protein and aligned using MAFFT. Maximum-likelihood reconstruction of the phylogeny of the superfamily was performed with FastTree2. (*B*) The F-box domain superfamily gene tree was reconciled with the chlamydial species tree to reconstruct the evolutionary history of this group for members of the *Chlamydiae*. The nodes in blue indicate the predicted number of F-box proteins, and numbers on the branches depict the gains and losses. The extant species are indicated with their respective counts for F-box proteins. The *Neochlamydia* have undergone massive gains and losses after the divergence from *Protochlamydia*.

Driven by the discovery that members of the four largest Parachlamydiaceae gene families showed rapid divergence and are kept in chlamydial genomes despite apparent functional redundancy, we asked whether there are additional genes not included in these gene families but encoding similar functional domains. The common theme of the large Parachlamydiaceae gene families is the presence of domains that serve in the recruitment of target proteins to the eukaryotic ubiquitination machinery. We therefore extracted all proteins containing F-box/F-box-like, BTB/POZ, and RING/U-box domains by scanning all chlamydial proteomes with each respective HMM profile. We found no RING/U-box containing proteins apart from those identified earlier as members of NEX1b, and we detected only few additional proteins in *Neochlamydia* and *Parachlamydia* harboring a BTB/POZ domain similar to those of PEX1. However, our search unveiled an astonishing number of proteins harboring F-box/F-box-like domains, with over 370 proteins within the phylum. Nearly 300 of the F-box proteins are the contribution of the two *Neochlamydia* species (129 in TUME1 and 158 in EPS4). To characterize the relationships among this F-box superfamily, we constructed a phylogenetic tree based on a domain alignment. This analysis shows that many of the additional F-box proteins found in *Neochlamydia* cluster with either NEX1a or NEX2 (fig. 5*a*). We also see several lineage-specific expansions of F-box proteins within *Protochlamydia* and *Parachlamydia* species. Reconciliation of the F-box superfamily tree with the chlamydial species tree confirms an extremely dynamic history of large-scale gene birth and death events (fig. 5*b*), mirroring the evolutionary pattern seen for the large Parachlamydiaceae gene families (supplementary figs. S2–S6 and table S3, Supplementary Material online).

Furthermore, we searched for additional chlamydial proteins containing domains for protein–protein interaction, such as LRR, TPR, or ankyrin repeats, which are often associated with F-box/F-box-like, BTB/POZ, and RING/U-box domains in the large gene families. This search identified a vast number of proteins for each domain. For instance, chlamydial genomes encode 409 proteins with LRR domains, nearly doubling the amount of LRR proteins contributed by the large Parachlamydiaceae gene families. Taken together, in addition to the four large gene families, there is an even greater pool of chlamydial proteins with a putative role in host interaction.

**Fig. 6.**
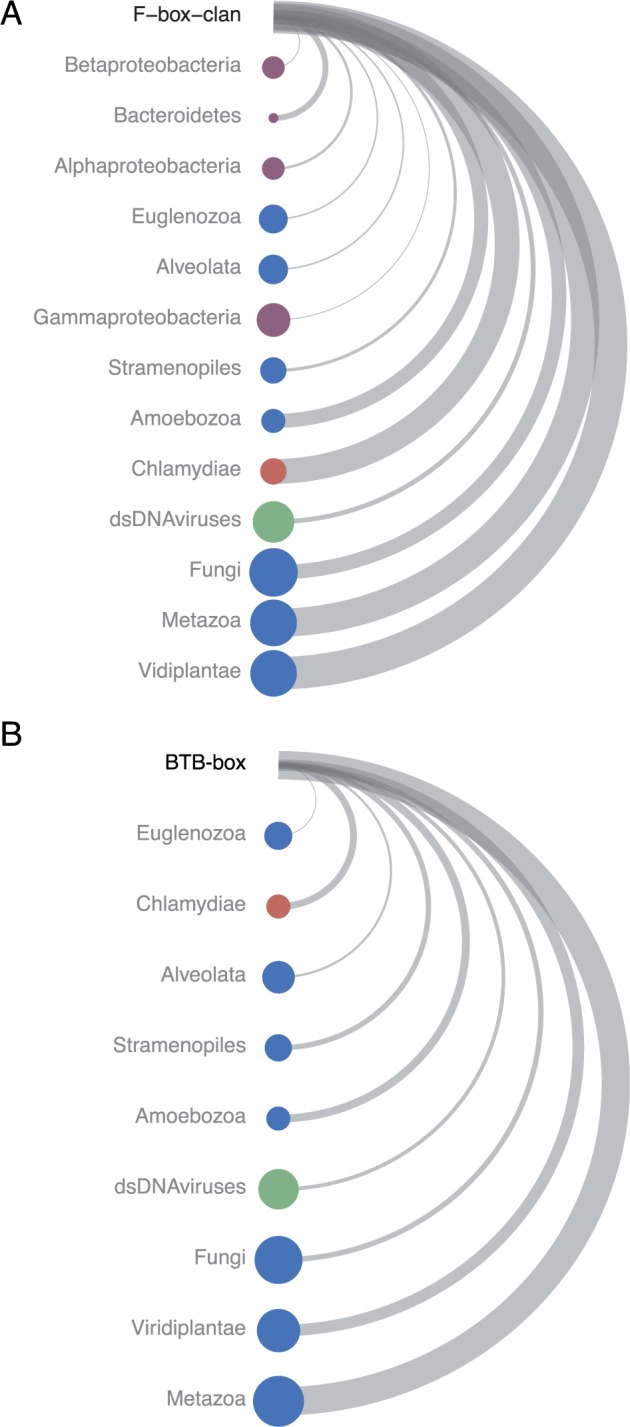
Taxonomic profile of F-box and BTB domains. The distribution of (*A*) the F-box clan, and (*B*) the BTB-box throughout sequenced organisms. The size of the node indicates the number of species harboring proteins with the domain. Thus, larger node size indicates a larger number of species in which a domain is found within a taxon. Nodes are ordered from least to greatest by the total number of proteins that contain the domain within the taxon. This is different than the number of species as one species can have many proteins harboring a given domain. To reflect this disparity and to facilitate comparisons, we computed a normalized value for each taxon that represents the number of total proteins divided by the number of species. This normalization value is represented by the width of the arc in the diagram. For instance, the chlamydiae are represented by few species (small node size) but are among the taxa containing the largest numbers of proteins with F-Box and BTB domains (position on vertical axis) and show a high number of proteins with these domains per species (arc width). All bacterial taxa are plotted in purple and selected major eukaryotic taxa in blue. The *Chlamydiae* are labeled in red, and double-stranded DNA viruses are shown in green. The data were obtained from the Pfam database for each domain, and counts were updated to reflect findings in this study.

To gain a broader overview of the occurrence of F-box/F-box-like and BTB/POZ domains among other prokaryotes and eukaryotes, we extracted domain abundance data from Pfam (Finn et al. 2013) and included the current counts for the *Chlamydiae* genomes present in this study (fig. 6). This revealed a striking pattern that the few bacterial groups encoding proteins with F-box and BTB domains are almost exclusively amoeba-associated organisms. These include members of the Legionellales (Gammaproteobacteria), the Rickettsiales (Alphaproteobacteria), and the amoeba symbiont *Amoebophilus asiaticus* (Bacteroidetes). When the number of F-box proteins is normalized against the total number of species in a given taxon, the *Chlamydiae* lead in the number of F-box proteins found in bacteria and even harbor more than several lineages of eukaryotes including the Amoebozoa. For the BTB proteins, the *Chlamydiae* appear to be the only bacterial lineage that harbors this domain. It is intriguing that many of the large double-stranded DNA viruses, namely the amoeba-infecting giant viruses, contain many proteins with an F-box or BTB domain.

## Discussion

### Large Gene Families Are Rare within Reduced Bacterial Genomes

Chlamydial genomes are among the smallest known for prokaryotes due to genome degradation consistent with long-term, obligate associations with eukaryotic organisms (McCutcheon and Moran 2011). The genomes of host-associated bacteria such as *Coxiella*, *Mycoplasma*, *Rickettsia* species, and members of the Chlamydiaceae, tend to have small or single copy, gene families (Gevers et al. 2004), and gene family expansions, either by gene duplication or HGT appear to have less effect on shaping the genomes of these obligate host-associated bacteria (Bordenstein and Reznikoff 2005). However, there is evidence that HGT may be more prevalent than once thought in these organisms (Blanc et al. 2007). A notable exception to this paradigm is the genome of the obligate intracellular pathogen *Orientia tsutsugamushi* (Rickettsiales), in which there has been massive expansion of type IV secretion system (over 350 *tra*-related genes) and host–microbe interaction genes (Cho et al. 2007). However, these expansions are thought to be the result of copious plasmid integration, and the genome is also littered with mobile elements, a scenario not shared within chlamydial genomes. Here, we have shown that several members of the *Chlamydiae* harbor gene families that have expanded at immense magnitudes, especially when compared with other intracellular bacteria (fig. 2). We find strong support for duplication processes contributing to the expansion of these families, thus highlighting that innovation through gene duplication has had pronounced effects in shaping these chlamydial genomes.

### Chlamydial Proteins Putatively Involved in Interference with Eukaryotic Ubiquitination Pathways

We demonstrated that the largest chlamydial gene families harbor proteins with domains associated with eukaryotic ubiquitination pathways and that the chlamydial F-box superfamily, in particular, is tremendous in size (figs. 3 and 5). A recent survey of prokaryotes found a total of 74 F-box proteins distributed in 22 species (Price and Kwaik 2010), which means the number of these proteins present in a single *Neochlamydia* genome is twice that of all previously known bacterial F-box proteins combined. The 76-member BTB superfamily is also remarkable in that the Parachlamydiaceae are the only prokaryotes known to harbor this domain (fig. 6).

In eukaryotes, ubiquitin plays a pivotal regulatory role as a posttranslational modification that includes targeting proteins for degradation. The process of adding ubiquitin to a protein occurs when an assembled E3 ubiquitin ligase complex carrying a target protein is bound to the ubiquitin conjugating enzyme E2. The ubiquitin ligase is a multiprotein complex termed the Skp1-Cullin-F-box protein complex, by which F-box proteins recruit target proteins and subsequently bind to Skp1, which is linked to a Cullin protein (Zheng et al. 2002). A RING/U-box protein then serves as a linker of E2 to the newly formed ubiquitin ligase, and the transfer of the ubiquitin moiety to the target occurs. BTB-box proteins can have multiple functions, but chief among them is a functional equivalent to the F-box protein in recruiting targets by binding to the E3 ubiquitin ligase complex (Perez-Torrado et al. 2006). Protein–protein interaction domains, such as ankyrin, kelch, WD-40, LRR, or TPR repeat domains, are coupled to F-box and BTB-box domains and confer the specificity for target proteins.

Given the conservation and essentiality of the ubiquitination pathway throughout eukaryotes, it should come as no surprise that bacterial pathogens have engineered ways to manipulate this pathway. The use of F-box proteins appears to be a common feature among plant pathogens, such as *Agrobacterium tumefaciens* and *Ralstonia solanacearum,* whose genomes encode one and four F-box proteins, respectively (Magori and Citovsky 2011). Intriguingly, F-box proteins seem to be a common feature of amoeba-associated bacteria and viruses. The amoeba symbiont *A. asiaticus*, a Bacteroidetes, is predicted to harbor 15 F-box proteins and until now was the largest known pool of these proteins among sequenced genomes (Schmitz-Esser et al. 2010). Additionally, *Legionella pneumophila* exports an F-box protein coupled to ankyrin repeats, termed AnkB, that is essential for infection of both human cell lines and *Acanthamoeba* (Price et al. 2009; Lomma et al. 2010)*.* AnkB blocks host proteosomal degradation and thus generates increased levels of required amino acids (Price et al. 2009; Price et al. 2011). Several other F-box proteins secreted by *L. pneumophila* have been shown to interact with host E3 ligase complexes (Ensminger and Isberg 2010). Among the members of the Chlamydiaceae, we could not detect an F-box or BTB-box domain. However, there are several proteins within the Chlamydiaceae that function as deubiquinating proteases, such as the *C. trachomatis* ChlaDub1 (Misaghi et al. 2006) and the recently described ChlaOTU characterized in *Chlamydia caviae* (Furtado et al. 2013).

We have shown experimentally that representative members of the investigated chlamydial gene families contain functional type III secretion signals (supplementary fig. S1, Supplementary Material online). Thus, they likely represent an extensive pool of effector proteins with a putative role in hijacking the host ubiquitination machinery, perhaps in a manner similar to AnkB from *Legionella*, by increasing nutrient availability. In the absence of direct protein–protein interaction data, however, we can only speculate as to what the interaction partner(s) are for the members of the PEX and NEX gene families.

### Birth-and-Death Evolution Has Shaped Large Parachlamydiaceae Gene Families

Large gene families are thought to generally either evolve via concerted evolution or according to a birth-and-death model (Nei and Rooney 2005). When gene families are evolving concertedly, all members experience the same evolutionary pressure and evolve as a unit. The gene family is marked by recombination between members that leads to a homogenization of all members, and thus in the phylogenetic analysis, one observes intraspecies clustering of gene family members. Although we do find minor evidence that recombination has occurred between members in the PEX and NEX gene families (supplementary fig. S8, Supplementary Material online), the effect does not appear to be that of homogenization. In contrast, long branch lengths in the trees and moderate overall sequence similarity indicate these proteins have diverged quite extensively (supplementary figs. S2–S6, Supplementary Material online). As we do not observe a dominance of intraspecies clustering in the phylogenetic analysis, these gene families are not evolving in a fashion as would be predicted via concerted evolution.

We provide clear evidence supporting birth-and-death evolution of the PEX and NEX gene families, which is marked by independent gains and losses of members (Nei and Rooney 2005). We detected frequent lineage-specific duplication and loss events, leading to high rates of variation in copy number between closely related organisms (supplementary table S3, Supplementary Material online). In the phylogenetic trees of the PEX and NEX gene families, we find interspecies clustering of members, which is a hallmark of the birth-and-death model (supplementary figs. S2–S6, Supplementary Material online). Additionally, we detected pseudogenized gene fragments of members related to the large gene families, which is another hallmark of this mode of evolution. To our knowledge, this mode of evolution has so far only been described once for a bacterial gene family (Rooney and Ward 2008).

It has been proposed that gene families that control phenotypic characters are generally subject to birth-and-death evolution (Nei and Rooney 2005; Nei 2007). In the case of the expansions seen in the Parachlamydiaceae, this character is likely the ability to effectively infect and replicate in protist hosts. The advantage of the birth-and-death scenario, as opposed to concerted evolution, is that individual members of the gene family are able to functionally differentiate from each other and thus might facilitate the adaptation to new ecological niches (Nei 2007). Therefore, a possible driver for the birth-and-death model for the PEX and NEX families is the exploitation of new ecological niches, which in this case is most likely new host(s) species or a novel way to subvert host cell machinery.

### A Confluence of Drift and Selection

Birth-and-death evolution of gene families occurs by both adaptive processes and chance events, such as genetic drift (Nei et al. 2008). This is due to the fact that, although gene duplications are intrinsically stochastic events, their fixation is influenced by both selection and genetic drift. If a duplicate is fixed, functional divergence can occur due to relaxed selection or diversifying selection in one of the gene copies, a process known as neofunctionalization (Lynch and Conery 2000). Once these genes have diverged, the new functions are then maintained in the genome via purifying selection. We envision an evolutionary scenario where the PEX and NEX gene family members rapidly diverged either due to positive diversifying or relaxed selection after duplication, likely leading to functional diversification. The window for detecting these early diversification processes is very small, but our analysis indicates that the gene family members are now being maintained via purifying selection.

The retention of such large gene families in organisms that typically undergo extreme genome reduction is perplexing, especially when considering the variation in copy number between organisms. The large number of gains and losses for F-box domain containing proteins across chlamydial lineages indicate substantial fluctuations in gene content, sometimes over short evolutionary time (fig. 5*b*). One described corollary of gene families evolving via a birth-and-death model is that the copy number variation of members within a gene family may vary due to chance duplication/loss events both between and within species, a process coined “genomic drift" (Nei et al. 2008). Genomic drift has been invoked to explain the large copy number variations in several large gene families in eukaryotes, including animal chemosensory receptors (Nozawa et al. 2007), homeobox genes (Nam and Nei 2005), and fatty-acid reductases (Eirín-López et al. 2012).

Remarkably, eukaryotic F-box and BTB-box gene families demonstrate the same dramatic evolution following a rapid birth-and-death model, and extensive copy number variation is seen both between and within eukaryotic species (Xu 2006; Navarro-Quezada et al. 2013). This is especially true in higher plant species (Stogios et al. 2005; Xu et al. 2009), where *Arabidopsis thaliana* and *Oryza sativa* harbor upwards of 800 F-box proteins (Xu et al. 2009), and large expansions have also been described in some nematode lineages (Thomas 2006). A case has been made that genomic drift also influences the evolution of these eukaryotic F-box/BTB-box protein families (Xu et al. 2009). Genomic drift therefore seems a plausible mechanism describing some aspects of evolution of the PEX and NEX gene families in the Parachlamydiaceae. Genome sequences from other chlamydial genomes and data from within populations would allow further testing of this hypothesis.

Another possibility is that the expansion of these families is the result of selection pressure due to a coevolutionary arms race with host counterpart protein(s). Under this scenario, the expansion and diversification of these families are in direct response to changes in target proteins. These evolutionary dynamics, also referred to as the Red Queen hypothesis (Valen 1974), have been most exemplified in bacteria within plant–pathogen relationships, most notably between the pathogen *Pseudomonas syringae* and its host *Ar. thaliana* (Ma et al. 2006; Baltrus et al. 2011). Copy number variation of effector proteins has been shown for *Ps. syringae*, and it is speculated that these differences confer differential host ranges (Baltrus et al. 2011). It is plausible, therefore, that variations in copy number between the PEX and NEX gene families is influenced by the host range of particular chlamydial lineages. This would imply that these proteins interact with many targets from a narrow host range or that there are a limited number of targets for a large number of possible hosts. It seems most parsimonious that the latter was the case and that these genes serve as accessory virulence factors allowing these chlamydiae to expand their host range. However, the failure to detect positive selection as the major force acting on the PEX and NEX gene family members casts doubts on a coevolutionary arms race as the sole mechanism for the evolution of these gene families.

Another conceivable scenario might be that selection pressure for increased gene dosage has contributed to the expansion of the large chlamydial gene families. In reduced genomes where the pool of regulatory proteins is limited, a path for increased protein expression might be gene duplication. This mode of “gene amplification" has been described, for instance, for the increase of antibiotic resistance genes in *E. coli*, the cholera toxin gene in *Vibrio cholerae*, and capsule biosynthesis genes in *Haemophilus influenza* (reviewed in Andersson and Hughes 2009). Although a gene dosage scenario cannot be fully ruled out for the PEX and NEX gene families, it seems unlikely as the high divergence of their members makes a completely overlapping function highly improbable. Additionally, gene amplifications are in response to strong selection pressure and would therefore be under strong positive selection. It is also known that once these selection pressures are removed, the amplified gene copies are rapidly lost within the population, often within several generations (Andersson and Hughes 2009).

We favor a hypothesis that incorporates both selection and chance into the equation. We envision that the expansion of these gene families is reflective of their role in host–microbe interaction, where they are interfering with the host ubiquitination pathway. The expansions may reflect either a change in the environmental niche, perhaps the ability to infect a new host organism, or they may be in response to a growing number of targets within a current host. Because the PEX and NEX gene families have, respectively, expanded in the *Protochlamydia* and *Neochlamydia*, they appear to provide lineage-specific functions related to particular host interactions. The PEX and NEX variation in copy number between closely related organisms may be reflective of genomic drift, in which the independent gain and loss of members within a family has been determined, to some extent, by chance events. The fact that members of the *Chlamydiae*, including pathogenic *Chlamydia*, also harbor various proteins to subvert the host ubiquitination pathway indicates a case of convergent evolution toward exploitation of this system within this phylum.

To summarize, the *Chlamydiae* harbor several lineage-specific gene families, which are the largest among intracellular microbes with small genomes. Experimental evidence and computational analysis strongly indicate that members of these large gene families function as effector proteins involved in manipulating the ubiquitination machinery of their eukaryotic host cells. The large chlamydial gene families follow a birth-and-death model of evolution, where genomic drift may influence copy number variation. This might represent a previously undescribed mechanism by which organisms with limited exposure to larger gene pools generate genetic diversity.

## Materials and Methods

### Genome Sequencing

We sequenced the genomes of *P. amoebophila* EI2 (Schmitz-Esser et al. 2008), *Parachlamydia acanthamoebae* OEW1 (Heinz et al. 2007), *Neochlamydia* sp. TUME1 (Fritsche et al. 2000), and *Neochlamydia* sp. EPS4. The *Acanthamoeba* sp. harboring the latter was isolated from pond sediment from Elba, Italy. Cells and DNA were prepared as described previously (Schmitz-Esser et al. 2010). All four genomes were sequenced using 454 technology, and assemblies were performed using Newbler 2.6. We implemented an in-house pipeline for genome annotation that combines multiple approaches for gene calling and function prediction. Gene calling was performed by combining ab initio predictions from GeneMark (Besemer et al. 2001), Glimmer v3.02 (Delcher et al. 2007), Prodigal (Hyatt et al. 2010), and Critica v1.05 (Badger and Olsen 1999) with homology information derived from a BLAST search against National Center for Biotechnology Information nonredundant protein database (NCBI Resource Coordinators 2014). RNA genes were called by tRNAscan-SE (Lowe and Eddy 1997), RNAmmer (Lagesen et al. 2007), and Rfam (Griffiths-Jones et al. 2005). Function prediction was performed via BLAST against the UniProt database (UniProt Consortium 2014), and domain prediction was performed via InterProScan 5 (Jones et al. 2014). Completeness estimates were performed based on the presence of single-copy marker genes (*n* = 54) found in 99% of all bacterial genomes. Sequences have been deposited at Genbank/EMBL/DDBJ under accession numbers PRJNA242498, PRJNA242497, PRJNA242499, and PRJNA242500.

### Comparative Genome Analysis

Orthologous protein groups were calculated using OrthoMCL (Li et al. 2003) with default parameters using the predicted proteomes from 19 chlamydial organisms (supplementary table S2, Supplementary Material online). For those gene families under analysis, membership to a given family was further evaluated by alignment and assessment of the phylogenetic trees. Members that had obvious major differences in the alignment and trees were dubbed spurious, and likely to have been grouped due to homology in the repeat region, and thus were removed from the gene family. Whole-genome alignments were performed using the MAUVE progressiveMauve algorithm (Darling et al. 2010). The MAUVE alignments and local synteny plots were visualized in R (v 3.0.1) with the genoPlotR package (Guy et al. 2010).

### Species Tree Construction

Phylogeny of the *Chlamydiae* was reconstructed using 32 phylogenetic marker proteins (supplementary table S2, Supplementary Material online). Multiple sequence alignments were performed with MAFFT (Katoh and Standley 2013) using the settings “–maxiterate 1000" and all alignments subsequently concatenated using FASconCAT v1.0 (Kück and Meusemann 2010). Maximum-likelihood analysis was performed with RAxML (Stamatakis 2006) with 1,000 bootstrap iterations under the PROTGAMMAGTR model, and Bayesian inference was performed with MrBayes v3.2 (Ronquist and Huelsenbeck 2003) using the mixed amino acid model and standard settings via the CIPRES science gateway (Miller et al. 2010). Alignment and tree files are available as supplementary material, Supplementary Material online.

### Gene Family Analysis

As multiple sequence alignments of repeat containing proteins are not trivial, we employed several methods to obtain reliable alignments. We compared the alignments produced by MUSCLE (Edgar 2004), MAFFT using the “genapairs" option (Katoh and Standley 2013), and DIALIGN-PFAM (Al Ait et al. 2013), all with and without trimming by Gblocks using relaxed parameters “-b5=h" (Castresana 2000). In nearly all cases, the MAFFT alignment combined with Gblocks yielded the best alignment as judged by manual inspection. The exceptions were that DIALIGN-PFAM with Gblocks was the best method for the NEX1b and PEX1 alignments. To ensure robustness, we calculated neighbor joining, maximum likelihood, and Bayesian trees for each of the alignment data sets and selected the most supported tree for the final analysis. In all cases, the Bayesian trees yielded the most support, which were calculated on the CIPRES gateway (Miller et al. 2010) with MrBayes as mentioned above for the species tree. For protein domain phylogenies, we extracted and aligned the domains using the hmmalign program from HMMER3 package (Eddy 2011), and phylogenetic trees for the domains were calculated using FastTree2 (Price et al. 2010). Reconciliations of gene trees to the species tree to infer gene gain and loss were performed in Notung v2.6 (Stolzer et al. 2012), and the root for the gene trees was assigned within the program to achieve the lowest duplication-to-loss ratio. Phylogenetic trees were visualized using iTOL (Letunic and Bork 2007) or the ETE2 toolkit (Huerta-Cepas et al. 2010). Alignment and tree files are available as supplementary material, Supplementary Material online.

### Detection of Selection and Recombination

Whole-protein alignments were converted to codon alignments via the Pal2Nal program (Suyama et al. 2006). To detect the mode of selection acting on these gene families, we used CodeML from the PAML package (Yang 2007). Only sequences that were nearly full length and not contig fusions were used for the CodeML analysis. We additionally used the modified Nei–Gojobori method for codon selection from the MEGA v5.1 (Tamura et al. 2011) program with 1,000 bootstraps and treating missing data with pairwise deletions. We used the RDP4 software suite (Martin et al. 2010) to detect recombination events, which is an amalgamation of many individual recombination programs linked into one software architecture. We employed seven recombination detection programs that include RDP (Martin and Rybicki 2000), GENECONV (Sawyer 1989), MaxChi (Smith 1992), BootScan (Martin et al. 2005), Chimaera (Posada and Crandall 2001), SiScan (Gibbs et al. 2000) and 3Seq (Boni et al. 2007). Only events that were predicted with more than four programs were considered.

### Intergenic Sequences Analysis

The proteome of *Protochlamydia* was used as query (tBLASTn) against the intergenic regions. The hits were conservatively filtered based on size (>50 aa), bitscore (>50), and *E* value (<10^−^^10^). For each intergenic region when two query proteins overlap, only the best hit was considered. In addition, if different parts of the same protein had hits in one intergenic space only, the top scoring (bitscore) was considered. Putative domains in the intergenic regions were detected using InterProScan 5 (Jones et al. 2014).

### Domain Distribution

For each domain of interest, we downloaded the Pfam HMM model (Finn et al. 2013) and scanned all chlamydial proteomes using the hmmscan program from the HMMER3 suite (Eddy 2011). In the case of the F-box and F-box-like domains, the results were combined into a nonredundant list. The data for the other taxa were obtained through the Pfam website under the species distribution tab for each domain or clan as in the case for the F-box. The networks were created using the “arcdiagram” package in R.

### Type III Secretion Analysis

Fusion proteins containing the 5′-part of the genes of interest (including the first 20 codons) and the adenylate cyclase Cya were expressed in *Sh. Flexneri* SF401 and SF620, derivatives of the wild-type strain M90T, in which the *mxiD* and *ipaB* genes have been inactivated (Allaoui et al. 1993). The 5′-part of the target genes were amplified by polymerase chain reaction and cloned in the puc19cya vector as described (Subtil et al. 2001). Secretion assays were performed on 30 ml of exponentially grown cultures as described previously (Subtil et al. 2001). Antibodies against CRP, a cytosolic marker, were used to estimate the contamination of supernatant fractions with bacterial proteins as a result of bacterial lysis. Antibodies against IpaD, a type-III-secreted protein of *Shigella*, were used to verify that type III secretion occurred normally in the transformed strains. A monoclonal antibody against Cya and polyclonal antibodies against CRP and IpaD were generously provided by Drs N. Guiso, A. Ullmann, and C. Parsot, respectively (Institut Pasteur, Paris). Prediction of type III secretion was performed via the Effective database (Jehl et al. 2011) and the PSORTb webserver (Yu et al. 2010).

## Supplementary Material

Supplementary tables S1–S3 and figures S1–S8 are available at *Molecular Biology and Evolution* online (http://www.mbe.oxfordjournals.org/).

Supplementary Data
