# Supplementary material for: Massive Expansion of Ubiquitination-Related Gene Families within the Chlamydiae
Source: Mol Biol Evol. 2014 Jul 28;31(11):2890–904. doi: 10.1093/molbev/msu227 (PMC4209131; doi:10.1093/molbev/msu227)
Supplement: Supplementary Data [file supp_31_11_2890__index.html]

Massive Expansion of Ubiquitination-Related Gene Families within the Chlamydiae — Massive Expansion of Ubiquitination-Related Gene Families within the Chlamydiae — Supplementary Data 

# Massive Expansion of Ubiquitination-Related Gene Families within the *Chlamydiae*

## Supplementary Data

files

**Files in this Data Supplement:**

- Supplementary Data - zip file
