# Supplementary material for: Massive Expansion of Ubiquitination-Related Gene Families within the Chlamydiae
Source: Mol Biol Evol. 2014 Jul 28;31(11):2890–904. doi: 10.1093/molbev/msu227 (PMC4209131; doi:10.1093/molbev/msu227)
Supplement: Supplementary Data [file supp_msu227_suppl_data.zip › Supplement_revision2.pdf]

Massive expansion of ubiquitination-related gene families within the *Chlamydiae*

Domman *et al*.

Supplementary Information

Table S1: Genome features of members of the *Parachlamydiaceae*

	***Protochlamydia amoebophila* UWE25**	***Protochlamydia amoebophila* EI2**	***Neochlamydia* sp. TUME1**	***Neochlamydia* sp. EPS4**	***Parachlamydia acanthamoeabae* OEW1**	***Parachlamydia acanthamoeabae* UV-7**
**Sequencing approach**	Sanger	454	454	454	454	Sanger
**Assembly**		Newbler 2.6	Newbler 2.6	Newbler 2.6	Newbler 2.6	
**Sequence length (nt)**	2,417,793	2,397,675	2,546,323	2,530,677	3,008,885	3,072,383
**Contigs >1 kb**	1	178	254	112	162	1
**Predicted CDSs**	2,031	2,150	2,345	2,174	2,756	2,788
**G+C content (%)**	35	35	38	38	39	39
**Coding regions (%)**	82	81	80	80	88	90
**Average CDSs length (nt)**	1,003	900	867	934	956	988
**tRNAs**	37	36	36	36	38	40

Table S2: Organisms used in this study and proteins used for species tree construction

Organism	Accession number	Genome size	Largest single expansion	Total expansions
***Chlamydia muridarum* Nigg**	NC_002620.2	1080453	4	10
***Chlamydia trachomatis* 434/Bu**	NC_010287.1	1038843	2	4
***Chlamydia trachomatis* D/UW-3/CX**	NC_000117.1	1042519	2	5
***Chlamydia trachomatis* L2c**	NC_015744.1	1038814	2	6
***Chlamydophila abortus* S26/3**	NC_004552.2	1144378	3	7
***Chlamydophila caviae* GPIC**	NC_003361.3	1181358	4	13
***Chlamydophila felis* Fe/C-56**	NC_007899.1	1173793	6	17
***Chlamydophila pecorum* E58**	NC_015408.1	1106198	6	13
***Chlamydophila pneumoniae* CWL029**	NC_000922.1	1230231	6	24
***Chlamydophila pneumoniae* LPCoLN**	NC_017285.1	1248552	9	23
***Chlamydophila psittaci* 6BC**	NC_015470.1	1179222	6	14
***Neochlamydia sp.* EPS4**	PRJNA242498	2530677	94	212
***Neochlamydia sp.* TUME1**	PRJNA242497	2546323	68	203
***Parachlamydia sp.* OEW-1**	PRJNA242499	3008885	8	92
***Parachlamydia acanthamoebae* UV-7**	NC_015702.1	3072383	12	92
***Protochlamydia amoebophila* UWE25**	NC_005861.1	2414465	41	154
***Protochlamydia amoebophila* EI2**	PRJNA242500	2397675	32	154
***Simkania negevensis* Z**	NC_015713.1	2496337	12	277
***Waddlia chondrophila* WSU 86-1044**	NC_014225.1	2116312	11	155

Ribosomal proteins		Other marker proteins
**Large subunit**	**Small subunit**	
rl1	rs3	RpoB
rl2	rs4	RpoC
rl3	rs5	GyrB
rl4	rs8	RecA
rl5	rs9	EfTu
rl9	rs15	
rl10	rs17	
rl11	rs18	
rl13	rs19	
rl14		
rl16		
rl20		
rl22		
rl23		
rl24		
rl27		
rl31		

Table S3. Gains and losses for large *Parachlamydiaceae* gene families. Events for each gene family after Notung reconciliation of the gene trees with the species tree are given as 'duplications/losses' for each organism.

	NEX1a	NEX1b	NEX2	PEX1	PEX2
**Proto UWE25**	NA	NA	NA	7/7	0/2
**Proto EI2**	NA	NA	NA	5/14	0/3
**Neo EPS4**	19/13	6/1	3/5	NA	NA
**Neo TUME1**	4/20	0/3	5/6	NA	NA
**Total Expansions**	78	12	25	49	14
**Total Losses**	33	4	11	21	5

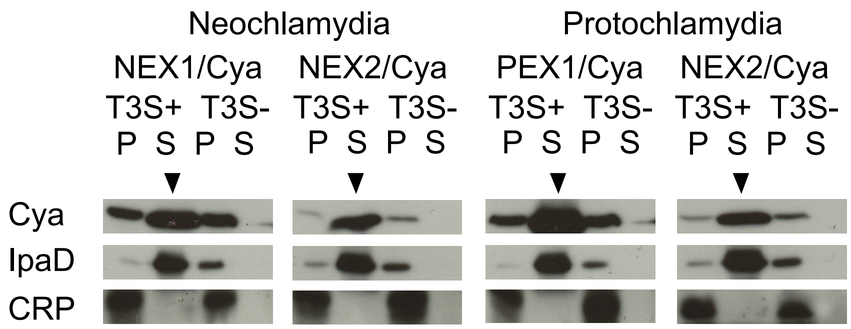

Supplementary Figure S1. **Type III secretion assay in *Shigella*.** A representative member from each of the gene families (NEX1: DB42_AK 00400; NEX2: DB42_CW00060; PEX1: YP_007742.1; PEX2: YP_007044.1) was tested for the presence of a functional type III secretion (T3S) signal in its N-terminus using *Shigella flexneri*. Chimera between the first 20 codons of the chlamydial genes and the reporter gene *cya* were transformed into a T3S competent strain (*ipaB*, T3S+) and a T3S deficient strain (*mxiD*, T3S-). Liquid cultures were fractionated into pellet (P) and supernatant (S), the protein extracts were run on a SDS-PAGE and transferred to a membrane. The membrane was probed with antibodies against IpaD, a known T3S substrate of *Shigella*, CRP, a cytosolic marker protein that serves as a control for cell lysis, and Cya. Each of the Cya chimeras tested was observed in the supernatant of the T3S+ strain (black triangles), and not of the T3S strain, demonstrating the presence of a functional T3S signal.

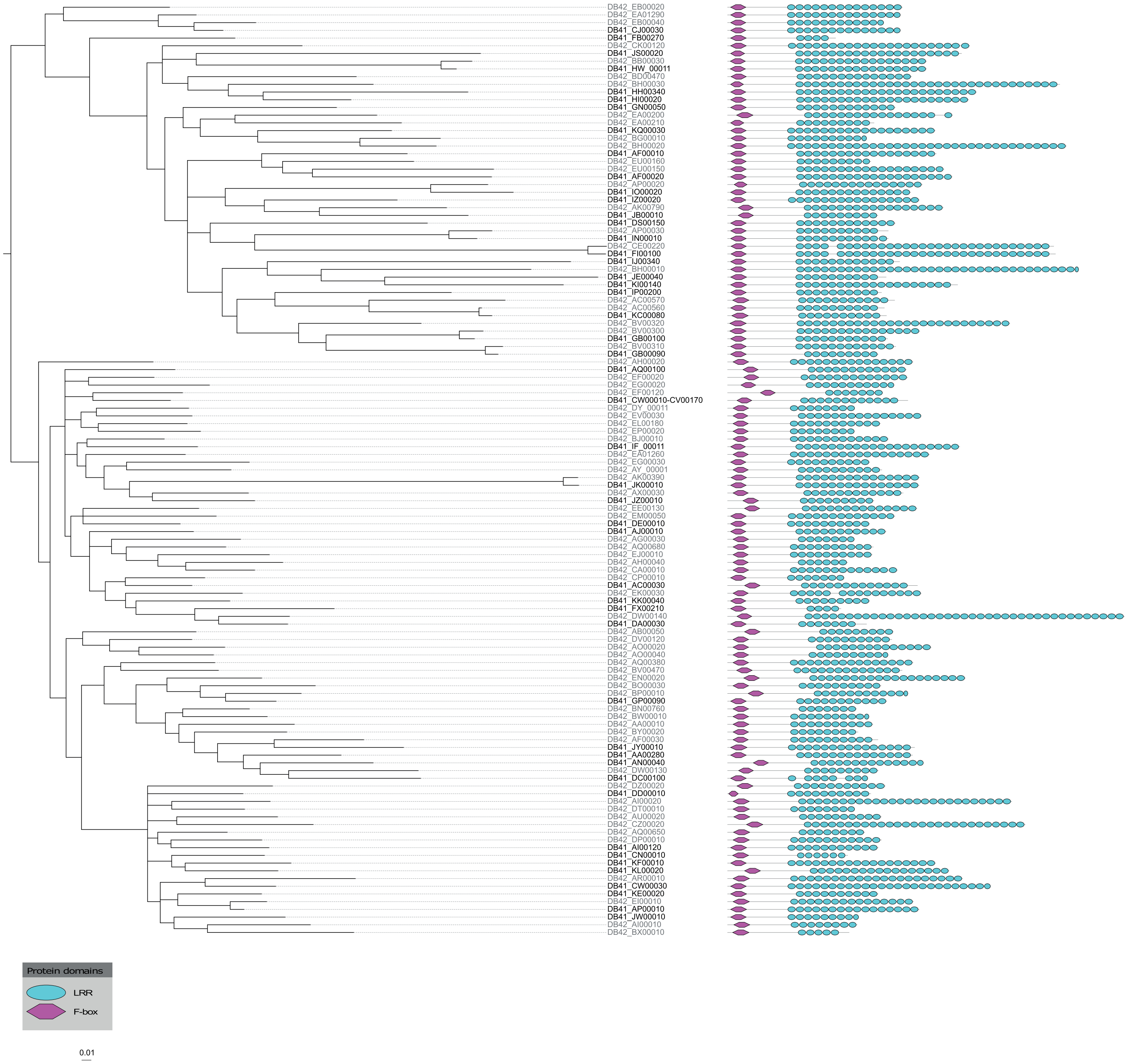

Supplementary Figure S2. **Phylogeny of the NEX1a gene family.**
A Bayesian reconstruction of the phylogenetic relationship between the NEX1a members is shown along with the corresponding protein domain architecture. There is a conserved F-box domain at the N-terminus followed by LRR domains. A region with no detectable domains between the F-box and LRR is conserved between the members. Shading in the sequence names differentiate between species, where *Neochlamydia* sp. TUME1 is colored in black and *Neochlamydia* sp. EPS4 is in grey.

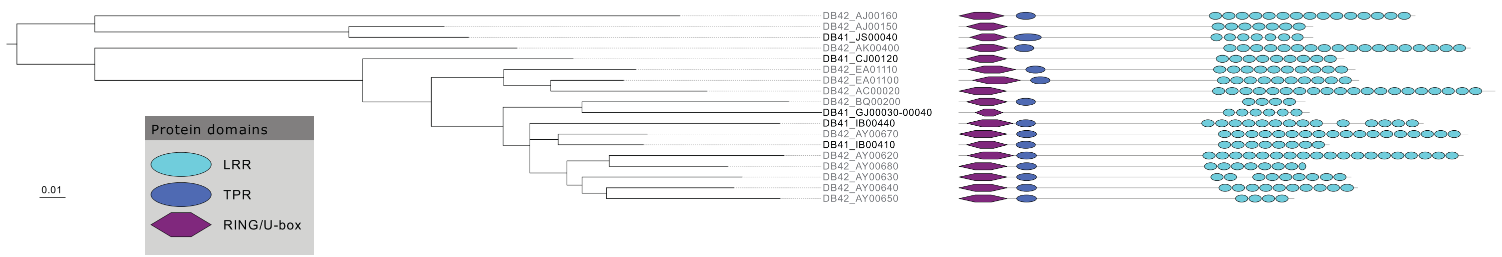

Supplementary Figure S3. **Phylogeny of the NEX1b gene family.**
A Bayesian reconstruction of the phylogenetic relationship between NEX1b members is shown along with the corresponding protein domain architecture. At the N-terminus there is a conserved RING/U-box domain followed immediately by a TPR domain. The C-terminus of the protein consists of various copies of LRR domains. Locus tags colored in black correspond to *Neochlamydia* sp. TUME1 and those in grey to *Neochlamydia* sp. EPS4.

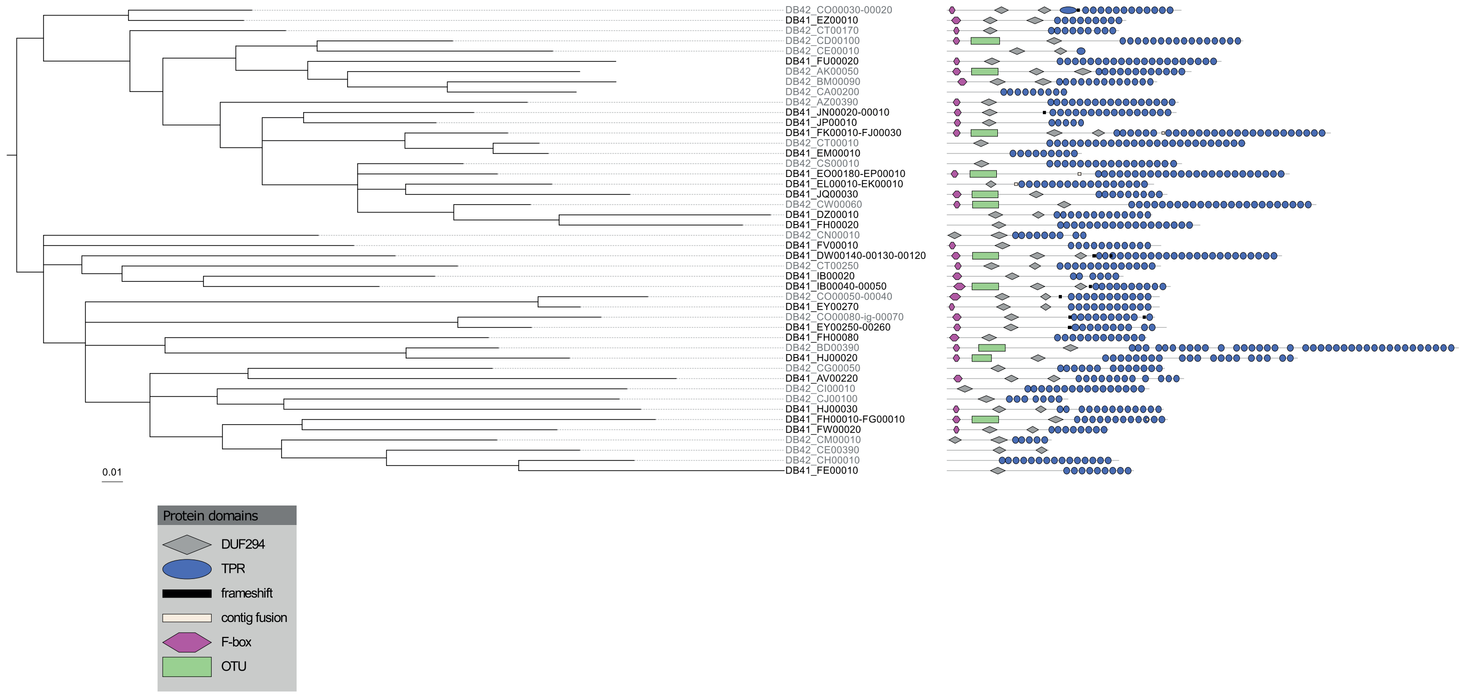

Supplementary Figure S4. **Phylogeny of the NEX2 gene family.**
A Bayesian reconstruction of the phylogenetic relationship between NEX2 members is shown along with the corresponding protein domain architecture. In the majority of members, there is an F-box domain at the N-terminus. In some cases (n=11) an OTU domain immediately follows. All members then harbor repeating DUF294 domains and a C-terminus of TPR domains. Contigs have been fused where indicated by a dash. Colors in the locus tags denote species differences where black and grey correspond to *Neochlamydia* sp. TUME1 and *Neochlamydia* sp. EPS4, respectively.

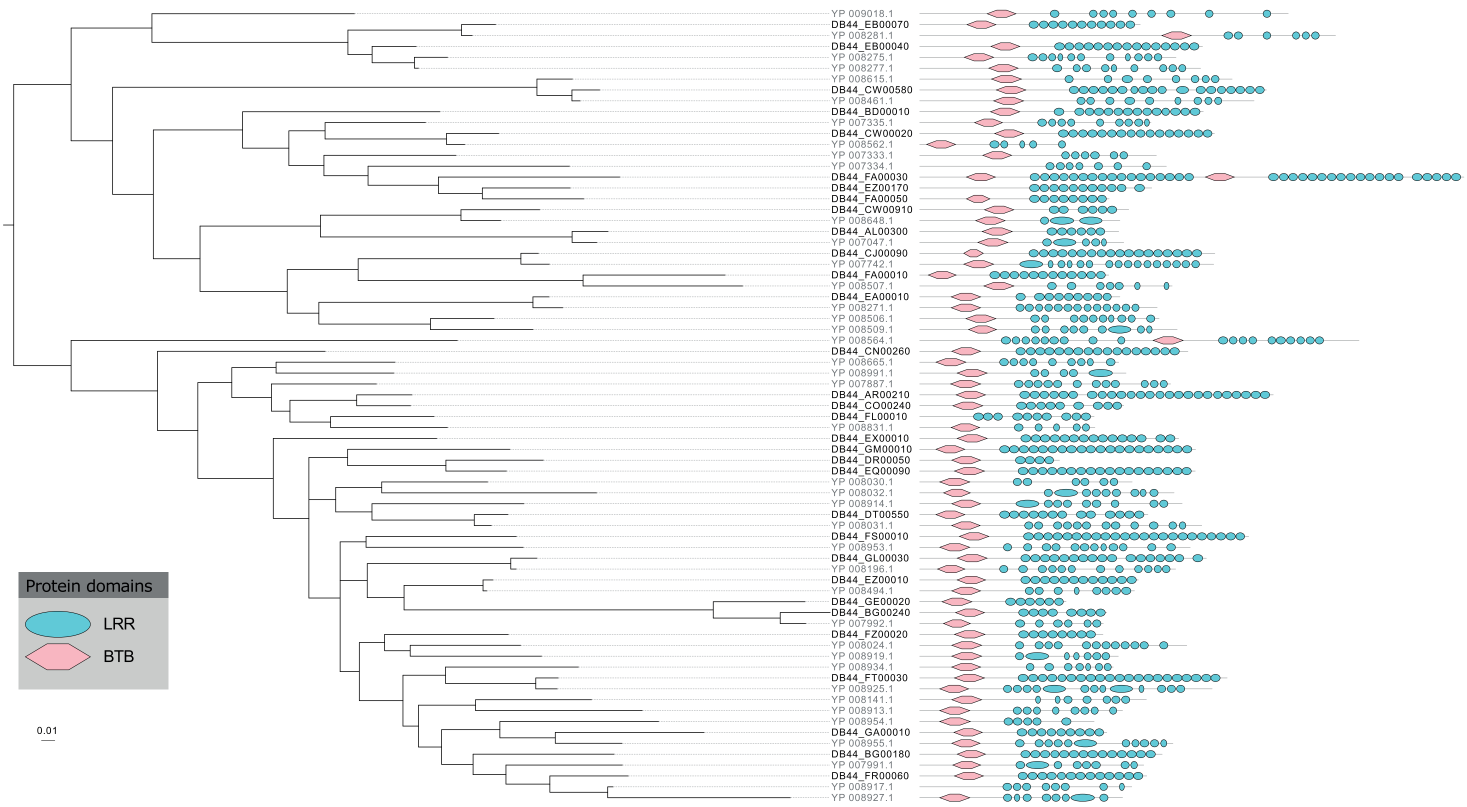

Supplementary Figure S5. **Phylogeny of the PEX1 gene family.**
A Bayesian reconstruction of the phylogenetic relationship between PEX1 members is shown along with the corresponding protein domain architecture. A BTB-box domain at the N-terminus is followed by multiple copies of LRR domains at the C-terminus. Locus tags colored in black correspond to *Protochlamydia amoebophila* EI2, while those in grey to *Protochlamydia ameobophila* UWE25.

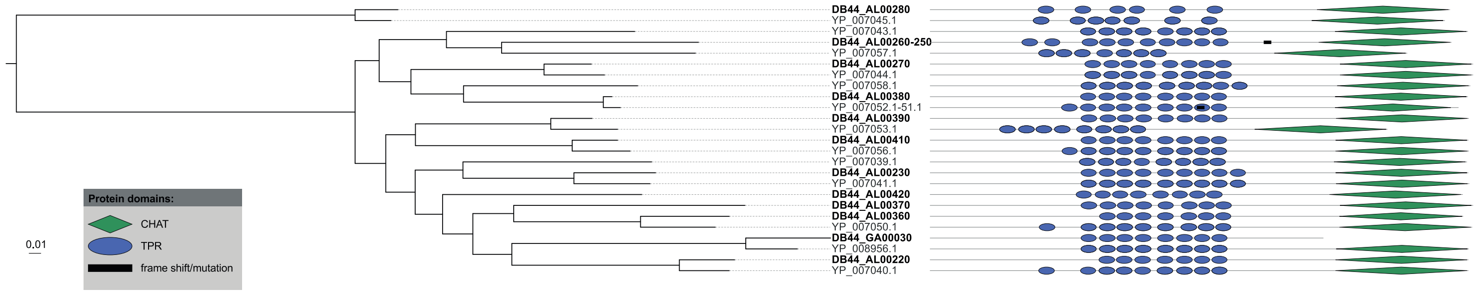

Supplementary Figure S6. **Phylogeny of the PEX2 gene family.** A Bayesian reconstruction of the phylogenetic relationship between PEX2 members is shown along with the corresponding protein domain architecture. There is no apparent N-terminal domain present in these members, but various copies of TPR domains appear in the middle of the protein. The C-terminus is marked by the presence of a CHAT domain. Black locus tags correspond to *Protochlamydia amoebophila* EI2, while grey locus tags are used for *Protochlamydia amoebophila* UWE25.

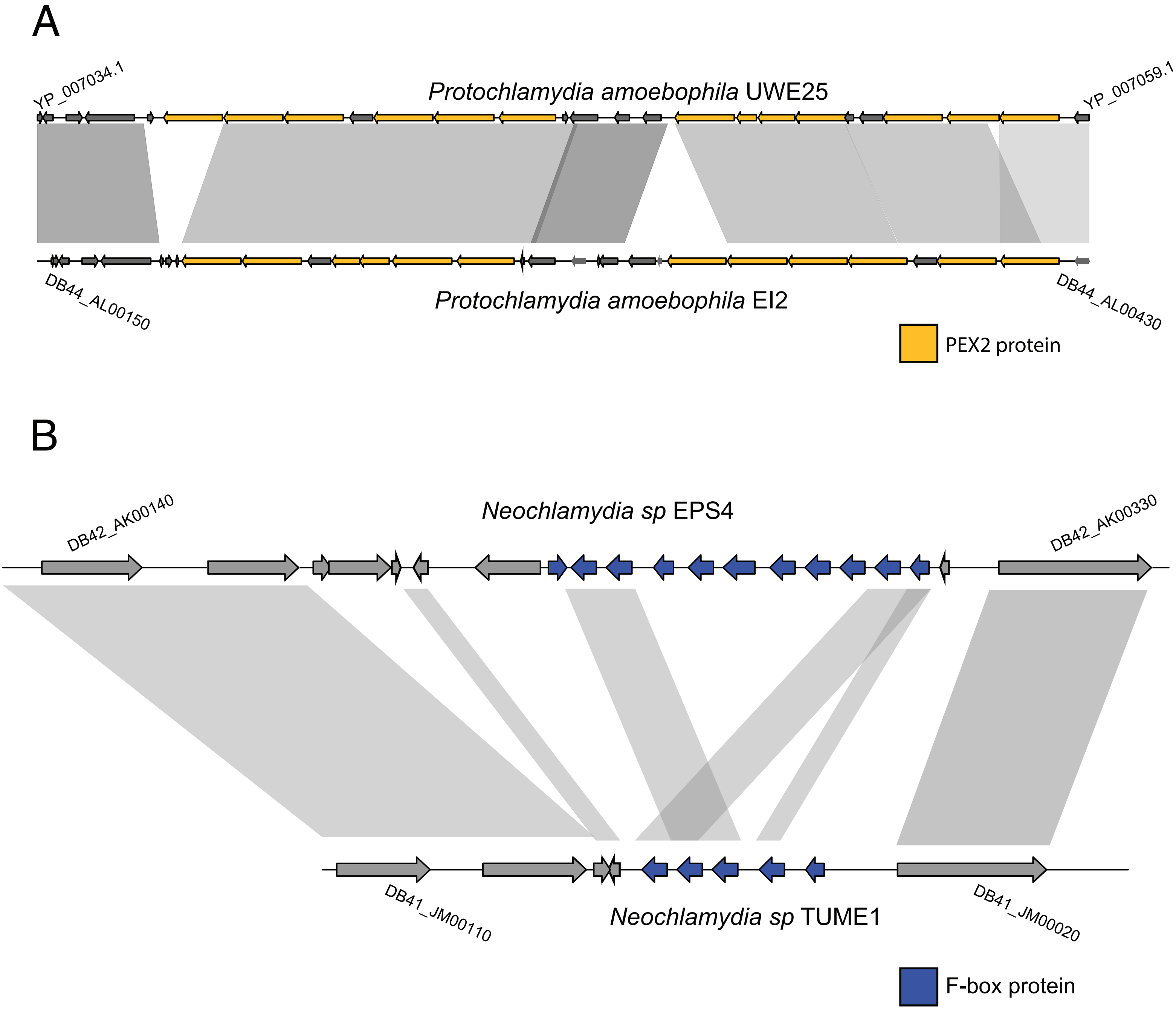

Supplementary Figure S7. **Evidence for tandem duplications among expanded *Parachlamydiaceae* gene families.** Members of the PEX2 gene family (A) in *Protochlamydia* and F-box proteins (B) in *Neochlamydia* are found in tandem arrays, which are strongly indicative of gene duplication events. Synteny between genomes is depicted with gray bars.

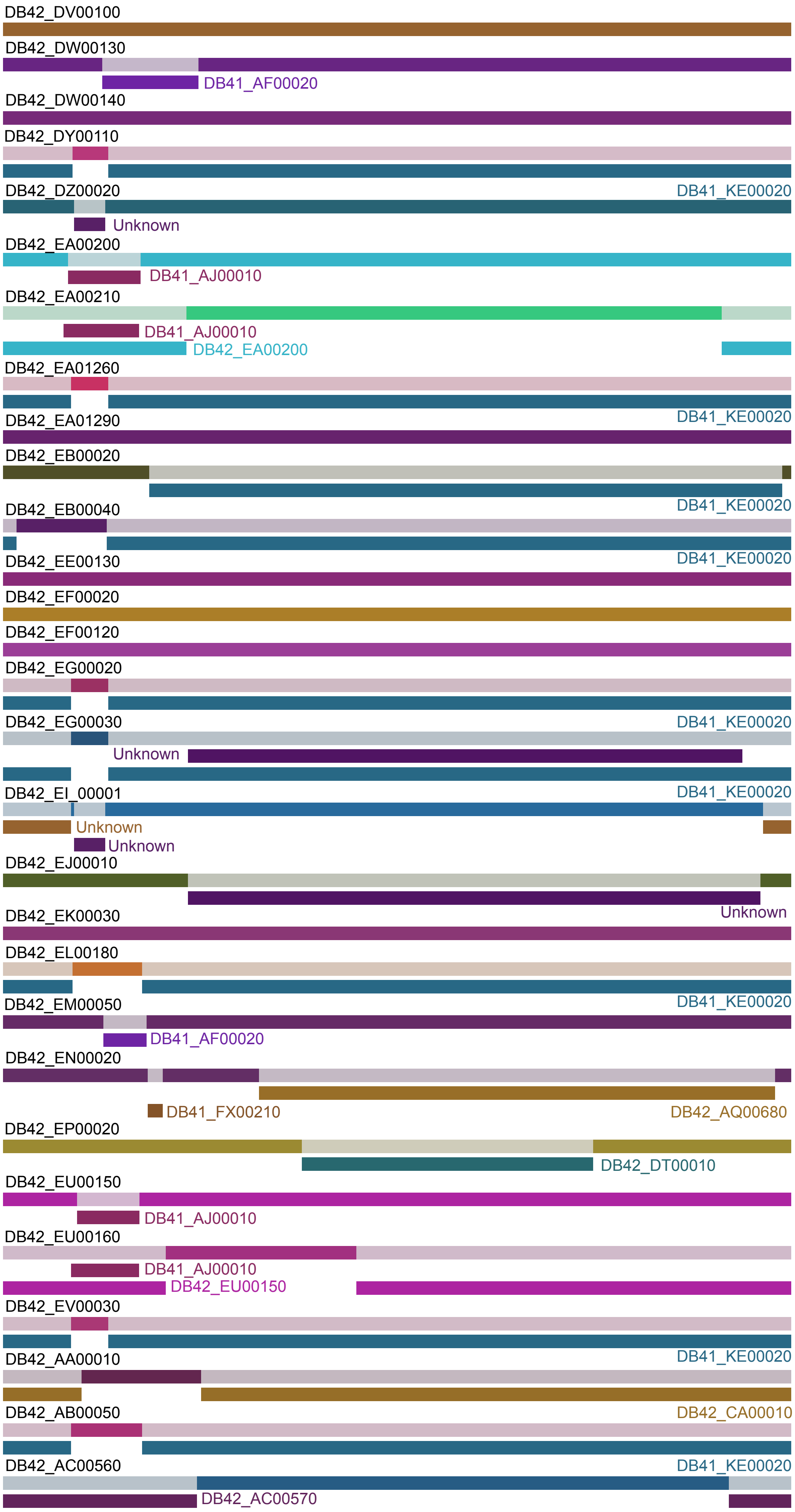

Supplementary Figure S8. **Example of recombination detection within the NEX1a gene family.** The output of the RDP4 program for detecting recombination in the NEX1a gene family is presented. Each sequence is represented by a full bar, with possible recombination points and the donor sequence shown below. Most of the predicted recombination regions are weakly significant. Note that many of the predicted recombination sites are in the vicinity of the N-terminal F-box-like domain.
